# Introgression of *Trifolium ambiguum* Into Allotetraploid White Clover (*Trifolium repens*) Using the Ancestral Parent *Trifolium occidentale* as a Bridging Species

**DOI:** 10.3389/fpls.2022.858714

**Published:** 2022-03-18

**Authors:** Ihsan Ullah, Helal A. Ansari, Isabelle M. Verry, Syed Wajid Hussain, Nick W. Ellison, Michael T. McManus, Warren M. Williams

**Affiliations:** ^1^AgResearch (Grasslands Research Centre), Palmerston North, New Zealand; ^2^College of Sciences, Massey University, Palmerston North, New Zealand

**Keywords:** interspecific hybridization, backcross breeding, genomic *in situ* hybridization, fluorescence *in situ* hybridization, bridge cross

## Abstract

White clover (*Trifolium repens*) is an allotetraploid pasture legume widely used in moist temperate climates, but its vulnerability to drought, grazing pressure and pests has restricted its wider use. A related species, Caucasian clover (*Trifolium ambiguum*), is a potential source of resistances to drought, cold, grazing pressure and pests that could potentially be transferred to white clover by interspecific hybridization. Although direct hybridization has been achieved with difficulty, the hybrids have not been easy to backcross for introgression breeding and no interspecific chromosome recombination has been demonstrated. The present work shows that interspecific recombination can be achieved by using *Trifolium occidentale*, one of the ancestral parents of *T. repens*, as a bridging species and that large white clover breeding populations carrying recombinant chromosomes can be generated. A 4*x* hybrid between *T. ambiguum* and *T. occidentale* was crossed with *T. repens* and then backcrossed for two generations. Five backcross hybrid plants with phenotypes appearing to combine traits from the parent species were selected for FISH-GISH analyses. Recombinant chromosome segments from *T. ambiguum* were found in all five plants, suggesting that recombination frequencies were significant and sufficient for introgression breeding. Despite early chromosome imbalances, the backcross populations were fertile and produced large numbers of seeds. These hybrids represent a major new resource for the breeding of novel resilient forms of white clover.

## Introduction

In this study, full sub-genomes (*x* = 8) are coded as follows: A from 4*x Trifolium ambiguum*, O from *Trifolium occidentale*, P^r^, O^r^ from *Trifolium repens*, representing the ancestral parent genomes from *Trifolium pallescens* and *T. occidentale*, respectively (Williams et al., 2012). R also from *T. repens* in cases where sub-genome origin is unclear. Partial sub-genomes are coded as the expected number of chromosomes (assuming normal segregation), e.g., O_4_ (four *T. occidentale* chromosomes).

Interspecific hybridization is a useful tool for the agronomic improvement of crops by enabling genetic diversity to be incorporated from wild and non-agronomic species. White clover, *Trifolium repens* L. (P^r^P^r^O^r^O^r^, 2*n* = 4*x* = 32), although an important pasture legume in moist temperate climates throughout the world, is vulnerable to stresses, including drought (Barbour et al., 1996; Brink and Pederson, 1998), grazing pressure (Forde et al., 1989), and pests (Alconero et al., 1986; Gaynor and Skipp, 1987; Latch and Skipp, 1987; Pederson and McLaughlin, 1989; Pederson and Windham, 1989). Breeding efforts for stress tolerances using the limited genetic variation within the primary gene pool have met with limited success (Williams et al., 2007).

*Trifolium ambiguum* M. Bieb. (Caucasian or Kura clover) is a rhizomatous species with resistance to drought, cold and pest attacks but also with several limitations that make it unsuitable for intensive grazing systems (Bryant, 1974; Spencer et al., 1975; Dear and Zorin, 1985; Mercer, 1988; Pederson and McLaughlin, 1989; Pederson and Windham, 1989; Sheaffer et al., 1992). However, it would be desirable if the resistance traits could be transferred to white clover by interspecific hybridization. Natural hybridization with white clover does not occur due to strong post hybridization barriers and, even with embryo rescue or ovule culture methods, very few fertile 4*x* hybrids have been produced by crossing 4*x T. ambiguum* (AAAA, 2*n* = 4*x* = 32) with *T. repens* (Williams and Verry, 1981; Meredith et al., 1995; Williams, 2014). Furthermore, when these AAP^r^O^r^ 4*x* hybrids were backcrossed to *T. repens*, most of the progeny were 6*x*, (AAP^r^P^r^O^r^O^r^) as the result of functional 2*n* gametes from the F_1_ hybrid (Anderson et al., 1991; Meredith et al., 1995), although rare 4*x* and aneuploid forms were also reported (Williams et al., 1982; Anderson et al., 1991). A second backcross produced 5*x* BC_2_ hybrids (AP^r^P^r^O^r^O^r^) with four sub-genomes (4*x* = 32) of *T. repens* and one sub-genome (*x* = 8) of *T. ambiguum* (Meredith et al., 1995). Interspecific chromosome pairing and recombination in these hybrids could potentially result in introgression and the transfer of *T. ambiguum* traits into *T. repens* by further backcrossing (Williams, 1987a; Meredith et al., 1995). However, such chromosome pairing occurred only at very low frequency (Williams et al., 1982; Anderson et al., 1991; Meredith et al., 1995) and to-date there has been no report of this strategy leading to introgression through interspecific meiotic recombination. Nevertheless, a breeding program using this backcrossing strategy was carried out in the United Kingdom, leading ultimately to cv. ‘Aberlasting’ (Abberton, 2007; Marshall et al., 2015).

Tetraploid (4*x*) *T. ambiguum* can also be crossed with a close relative and progenitor of white clover, *T. occidentale*, using embryo rescue. When colchicine-doubled 4*x T. occidentale* (OOOO) was used, the 4*x* (AAOO, *T. ambiguum* × *T. occidentale*) F_1_ hybrids were fertile and showed evidence of frequent interspecific meiotic chromosome pairing (Williams et al., 2019a). In addition, the hybrids were able to be crossed to white clover to generate tri-species (P^r^O^r^AO) hybrids (Williams et al., 2019a). The knowledge that white clover is an allotetraploid and that one of its ancestral sub-genomes was from *T. occidentale* (Ellison et al., 2006; Williams et al., 2012), led to the suggestion of using *T. occidentale* as a species bridge to achieve introgression of *T. ambiguum* into white clover. The rationale is that interspecies recombination in *T. ambiguum* × *T. occidentale* hybrids would create recombinant chromosomes having *T. occidentale* centromeres with *T. ambiguum* genomic segments on the arms, and vice versa. Then crossing of these hybrids, having recombinant chromosomes with *T. repens*, followed by further backcrossing to *T. repens* might lead to the introgression of *T. ambiguum* genome segments into *T. repens* genomes as a result of the substitution of, or meiotic exchange between, the *T. occidentale* chromosomes from both parents (Williams, 2014; Williams et al., 2019a).

**Figure 1:**
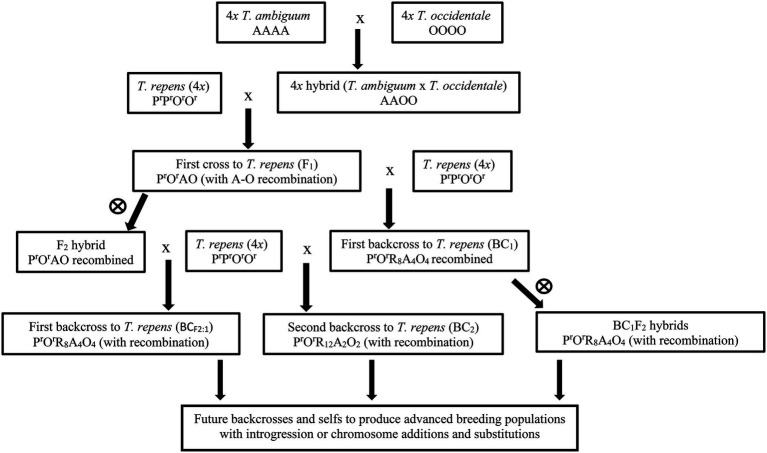
Flow chart of the hybridization and breeding program. A, O, P^r^, O^r^ represent haploid sub-genomes (*x* = 8) of *Trifolium ambiguum*, *Trifolium occidentale*, ancestral *Trifolium pallescens* from *Trifolium repens*, ancestral *T. occidentale* from *T. repens*, respectively. R represents chromosomes from *T. repens* where the sub-genomes could not be defined. Partial sub-genomes are designated by subscripts, e.g., O_4_ represents four *T. occidentale* chromosomes. Numbers are those expected assuming random assortment. Female parents are generally on the left side, with one exception (designated ♂). BC_1_ families were produced using crosses in either direction (designated ♂♀). 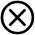 indicates self-pollination.

The proposed introgression breeding strategy (Figure 1) would start with 4*x* tri-species *T. repens* × (*T. ambiguum* × *T. occidentale*) hybrids, derived from crosses between white clover plants and 4*x T. ambiguum* × *T. occidentale* hybrids. These would be backcrossed to white clover and the progenies screened for desired *T. ambiguum* traits. Selected plants would then be further backcrossed to white clover and the process completed until new forms of white clover carrying introgressed *T. ambiguum* characteristics were produced.

Here, we test the feasibility of this strategy by carrying out a hybridization and backcrossing program starting with a tri-species hybrid and backcrossing it to white clover. Advanced families were screened for *T. ambiguum* traits and for chromosome pairing and genomic exchange using both conventional and molecular (FISH-GISH) cytogenetics. The frequent incorporation of chromosome segments from 4*x T. ambiguum* into hybrids with *T. repens* by meiotic recombination is reported for the first time.

## Materials and Methods

The starting materials for the study (Figure 1) were first crosses (F_1_) between a *T. repens* plant (Red-1) and a 4*x T. ambiguum* × 4*x T. occidentale* hybrid (BL-1), with the expected genomic composition P^r^O^r^AO, as described by Williams et al. (2019a) and listed in Table 1.

**Table 1:** Flow cytometric estimates of ploidies of 4*x* F_1_ (Red-1 × BL-1) hybrids [*Trifolium repens* × (4*x Trifolium ambiguum* × 4*x Trifolium occidentale*)], with the expected genomic composition P^r^O^r^AO.

Hybrid	Pedigree	Estimated ploidy (*x*)
F_1_-119	(Red-1 × BL-1)-1	3.9
F_1_-123	(Red-1 × BL-1)-6	3.8
F_1_-124	(Red-1 × BL-1)-7	3.6
F_1_-125	(Red-1 × BL-1)-8	3.9
F_1_-136	(Red-1 × BL-1)-19	3.9

### Plant Hybridization and Selection Procedures

Seeds were scarified with sandpaper and germinated on moist filter paper in Petri dishes at room temperature. The seedlings were planted in plastic pots containing potting mix (peat and sand in equal ratio) in a greenhouse under natural daylight without heating. Plants were grown in an insect-free unheated glasshouse at the AgResearch Grasslands Research Centre, Palmerston North, New Zealand. The plants were watered as needed and fed once fortnightly in solution form with a commercially available complete nutrient solution (Yates Thrive®).

Crosses were made after forceps emasculation (Williams, 1954) of the female parent by brushing anthers of the male parent over the exposed stigmas. This was usually repeated daily for the subsequent 2–3 days. Self-pollination was done by gently rolling inflorescences between thumb and fingers and repeating over 2–3 days. Dried inflorescences (heads) were harvested and threshed between two pieces of corrugated rubber. Self-compatibility of all plants used as female parents was determined by using the simple techniques suggested by Williams (1987b). To estimate male fertility, anthers were squashed on a glass slide and the pollen stained in 2% (w/v) aceto-carmine. The percentage of full stained pollen grains was recorded among a minimum of 300 counted at 200x magnification.

Selection of plants for evaluation and crossing was based on vigour, flow cytometric determinations of ploidy, somatic chromosome counts and above-ground appearance combining characteristics from the parent species. Ploidy analysis was based on DNA content using flow cytometry using the procedure described by Williams et al. (2011). For the above-ground appearance, emphasis was placed on *T. repens*-like plants with the additional appearance of one or more *T. ambiguum* characteristics – sometimes coarser or more elongated leaflets, determinate stems with weak nodal rooting and thick root bases visible at the soil surface.

The F_1_ plants were backcrossed to white clover plants to produce BC_1_ families and were also inter-crossed or self-pollinated to produce F_2_ families (Figure 1; Table 1). One hundred seeds from the crosses to white clover were germinated and the seedlings grown in pots in the greenhouse. Among the most robust survivors, 19 were subjected to further fertility and ploidy analyses. From these, six plants (five BC_1_ and one F_2_, Table 2) were selected for detailed characterization and backcrossing based on their vigour, fertility, estimated chromosome number and above-ground appearance combining characteristics from the parent species. Subsequently, the BC_1_ plants were backcrossed to *T. repens* to produce BC_2_ families (Figure 1) and were also self-pollinated to give BC_1_F_2_ families (Figure 1). The F_2_ plants were crossed with *T. repens* to produce BC_1_ families, here designated BC_F2:1_ to indicate derivation from F_2_ (Figure 1). Approximately 10 plants of each of these advanced families were grown in pots in the glasshouse and plants (Table 3) selected as before underwent further analysis.

**Table 2:** BC_1_ and F_2_ plants selected for detailed analysis, including actual chromosome numbers, pollen fertility, seeds obtained per inflorescence (approx. 50 florets) when crossed with *T. repens*, and whether self-compatible (SC) or self-incompatible (SI).

Plant	Parentage (♀ × ♂)	Actual 2*n* number	Pollen staining (%)	Seeds/infl.	SC/SI
BC_1_-120	(PB-5 × F_1_-123)-1	33	43	7	SC
BC_1_-128	(F_1_-119 × PB-5)-1	31	30	69	SC
BC_1_-130	(F_1_-119 × PB-5)-2	31	67	66	SC
BC_1_-131	(F_1_-119 × C2418-2)-1	34	58	23	SC
BC_1_-132	(F_1_-136 × PB-17)-1	33	66	29	SC
F_2_-133	F_1_-119 self-5	35	43	3	SI

*PB white clover plants were purple leaved*.

**Table 3:** Selected BC_2_, BC_F2:1_ and BC_1_F_2_ progeny with expected and actual chromosome numbers and pollen fertilities.

Hybrid	Pedigree	Expected 2*n* number	Actual 2*n* number	Pollen staining (%)
BC_2_-126	(Kopu II-906 × BC_1_-120)-1	32–33	33	51
BC_2_-129	(Kopu II-901 × BC_1_-128)-10	31–32	32	94
BC_2_-131	(Kopu II-910 × BC_1_-130)-4	31–32	32	70
BC_F2:1_–136	(Kopu II-918 × F_2_-133)-5	32–35	35	32
BC_1_F_2_–137	BC_1_-128-Self-1	30–32	32	62
BC_1_F_2_–138	BC_1_-131-Self-8	34	34	61
BC_1_F_2_–140	BC_1_-132-Self-3	32–34	32	52

Most of the white clover plants used as male parents carried co-dominant purple anthocyanin leaf color markers, whereas those used as females were usually unmarked (i.e., green). C numbers relate to accessions from the Margot Forde Germplasm Centre, Palmerston North.

### DNA Analysis, Cytology and Molecular Cytogenetics

The procedures were carried out in two separate experiments. In the first, root tip cells and meiotic PMCs from BC_2_-126 were subjected to FISH-GISH using 5S rDNA and total genomic DNA from *T. ambiguum*. The results showed evidence of recombination and so four more plants were sampled. These included two BC_1_ plants (BC_1_-120, BC_1_-132) and two BC_2_ plants (Kopu II × BC_1_-132, BC_2_-133) which underwent FISH using 5S and 18S rDNA followed by sequential FISH-GISH with the centromeric probe TrR350 (Ansari et al., 2004) and *T. ambiguum* total genomic DNA. The methods for somatic chromosome preparations, Giemsa staining, DNA preparation and FISH using 5S and 18S rDNA probes were as described by Ansari et al. (1999). DNA preparation and FISH using TrR350 were as described by Ansari et al. (2004). The GISH procedures were described in Ansari et al. (2008). Total genomic DNA of *T. ambiguum* was extracted from 2*x* cv. Summit. Before meiotic GISH preparations were implemented, an additional step of pepsin treatment was included to remove the proteinaceous background caused by the dense cytoplasm of pollen mother cells (PMCs). Two plants (BC_1_-120, BC_2_-126) were analyzed for meiotic metaphase I configurations using conventional cytology as described by Williams et al. (2011).

### Hybrid Plant Phenotypes

After their selection based on the characteristics described above, the plants listed in Tables 2, 3 were clonally propagated as cuttings and grown in randomized field trials and measured for a range of phenotypic traits. The methods and results are provided as Supplementary Material. Because they were done later, the descriptions did not influence the selection process. However, they did give retrospective indications of the variability of the hybrids in vigour, fertility and the extent to which they had combined traits from the parent species.

## Results

Flow cytometry estimates indicated that the five starting P^r^O^r^AO parent plants were all near-4*x* (Table 1). Two plants, F_1_-125 and (Red-1 × BL-1)-9 (the latter not used in crosses) were checked for chromosome numbers and were both 2*n* = 32 with the expected three satellited chromosomes (one from each of *T. ambiguum*, *T. occidentale*, and *T. repens*).

Following the crosses shown in Table 1, five BC_1_ plants were selected for further analysis (Table 2). The expected genomic composition of these plants was P^r^O^r^R(A_4_O_4_) (32 chromosomes), including partial genomes from *T. ambiguum* and *T. occidentale*. One of the *T. repens*-derived genomes (designated R) was expected to be an unspecified mixture of chromosomes derived from both sub-genomes of white clover. The Giemsa-stained somatic chromosome counts in these hybrids ranged from 31 to 34. Also selected for analysis was F_2_-133, which had 35 chromosomes, and was derived from a self-pollination. Pollen staining results (30–67%) indicated low-medium male fertilities. These plants were self-pollinated and, as they had leaf color markers, they were also used as male parents to pollinate a totally green *T. repens* plant (Kopu-II, a commercial white clover variety). All these selected plants produced seed when crossed with white clover (Table 2). All except F_2_-133 also proved to be self-compatible, setting large quantities of seed following self-pollination.

The resulting BC_2_ and BC_1_F_2_ progeny of 63 plants from a mixture of backcrosses and selfs were grown in the greenhouse and, using plant phenotypes, including leaf color markers, seven plants were selected for further analysis (Table 3). The expected genomic formula in the three backcrosses, BC_2_-126, −129 and-131, was P^r^O^r^(R_12_A_2_O_2_) and chromosome counts of 33, 32 and 32, respectively, all were within the range of expectations. BC_F2:1_–136 resulted from the backcross of F_2_-133 (35 chromosomes) with white clover. The female gamete from white clover in this case was fertilized by a male gamete from F_2_-133 having 19 chromosomes. BC_1_F_2_–137, −138 and − 140 were the respective self-progeny of BC_1_-128, −131 and − 132 and had somatic chromosome numbers of 32, 34 and 32, within the expected ranges (Table 3). Male fertility in all these hybrids was above 50%, except for BC_F2:1_–136 (32%).

### Molecular Cytogenetic Analysis of BC_2_-126 Somatic Cells

**Figure 2:**
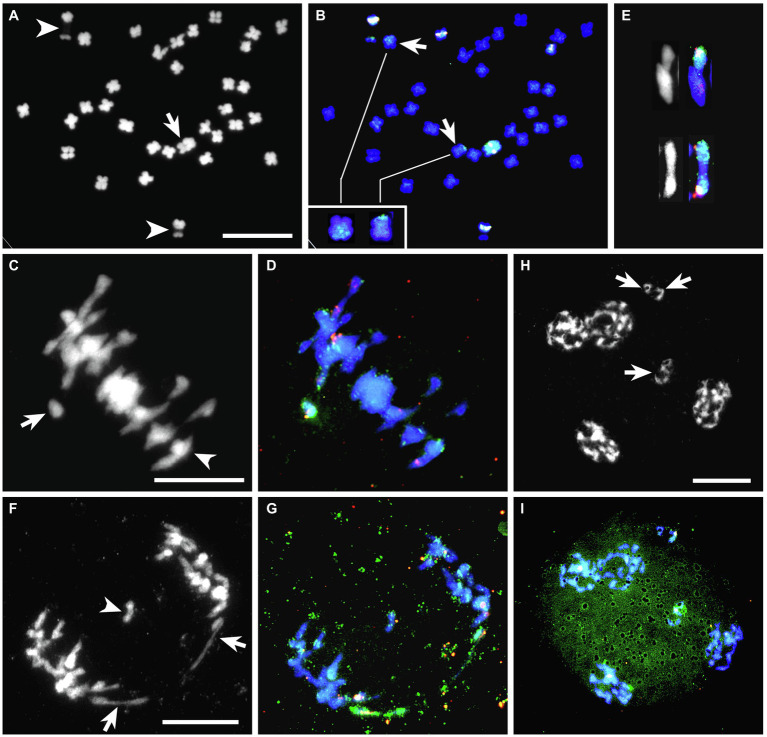
BC_2_-126 sequential FISH-GISH results with 5S rDNA (pink, white) and *T. ambiguum* genomic DNA (green). **(A,B)**, A somatic cell **(A)** DAPI stained (gray scale) and **(B)** the same cell after FISH-GISH. In **(A)**, the *arrow* depicts a single *T. ambiguum* chromosome, and *arrowheads* show NOR-chromosomes from *T. repens* or *T. occidentale*. In **(B)**, the *arrows* show recombinant chromosomes, which are magnified in the inset. **(C,D)**, A meiotic metaphase I (MI) cell with the same probes as in **(A,B)**. In **(C)**, the arrow shows the unpaired *T. ambiguum* chromosome as a univalent and the arrowhead marks a trivalent involving two recombinant chromosomes and a 5S rDNA-bearing chromosome from *T. repens* or *T. occidentale*. **(E)**, Cut-outs from other MI cells of a trivalent (top) and a bivalent (bottom) involving *T. ambiguum* or recombinant chromosomes. **(F,G)**, Anaphase I cell with the same probes as in **(A,B)**. In **(F)**, the *arrows* show the two halves of the *T. ambiguum* chromosome which has undergone precocious separation of the sister chromatids (PSSC) and the *arrowhead* marks a laggard chromosome. **(H,I)**, A telophase II with the same probes as in **(A,B)**. In **(H)**, the *arrows* mark micronuclei with chromosomes likely to be eliminated from the gametes. In **(I)**, the elimination of the lone *T. ambiguum* chromosome is shown. Bars 10 μm.

A BC_2_ hybrid, BC_2_-126 with 33 chromosomes, was derived from a backcross of BC_1_-120 to white clover. DAPI staining of somatic cells of BC_2_-126 revealed the presence of one very large chromosome and two chromosomes with decondensed nucleolus organizer regions (NOR; Figure 2A). GISH using labeled genomic DNA from *T. ambiguum* (green) painted the very large chromosome, indicating that it was *T. ambiguum*-derived (Figures 2A,B). GISH consistently also revealed recombination between *T. ambiguum* chromosomes and two *T. repens* or *T. occidentale*-derived chromosomes. This manifested as one large and one smaller green signal on two chromosomes (Figure 2B). This hybrid gave six signals when probed with 5S rDNA. Two 5S signals were on a pair of *T. repens* or *T. occidentale* chromosomes bearing de-condensed NOR sequences and three were on non-NOR *T. repens* or *T. occidentale* chromosomes. The *T. ambiguum*-derived chromosome also carried a 5S rDNA signal (Figures 2A,B).

### Chromosome Pairing Analysis in BC_2_-126

In conventionally stained metaphase I cells of BC_2_-126 (2*n* = 33) chromosome pairing was highly variable from cell to cell (Table 4). On average, approximately 73% of the chromosomes paired as bivalents with the remainder as univalents and multivalents. Sequential FISH-GISH analysis was carried out on meiotic chromosome preparations of BC_2_-126 using labeled genomic DNA from *T. ambiguum* (green) and 5S rRNA (red). Among 21 PMCs analyzed at metaphase I, 9 (43%), had the *T. ambiguum-*derived chromosome unpaired (Figures 2C,D) while in the remaining 12 cells this chromosome paired with chromosomes from either white clover or *T. occidentale*. In six of these the *T. ambiguum* chromosome associated as a bivalent and in six it paired to form multivalents. The recombinant chromosomes also paired in bivalents or multivalents with *T. repens* or *T. occidentale* chromosomes (Figure 2E).

**Table 4:** Meiotic chromosome associations at diakinesis/metaphase I in PMCs of BC_1_-120 (2*n* = 33) and BC_2_-126 (2*n* = 33).

Plant	No PMCs	Mean frequency (range) of meiotic configurations					
		I	II	III	IV	V	Pollen stain (%)
BC_1_-120	35	2.5 (0–7)	9.2 (5–15)	2.0 (0–5)	1.3 (0–4)	0.1 (0–1)	43
BC_2_-126	47	3.5 (0–9)	12.0 (6–15)	1.1 (0–4)	0.6 (0–2)	0	51

At anaphase I, univalents frequently showed precocious separation of sister chromatids, followed by movement to opposite poles. This is illustrated for a *T. ambiguum* chromosome in Figures 2F,G where chromatid separation was clear because this chromosome also had a 5S red signal and, after splitting, the two 5S signals could be seen moving to opposite poles (Figure 2G), or sometimes lagging behind. A laggard chromosome is shown in Figures 2F,G. In many cases at telophase II, the lagging chromatids did not become part of the tetrads but formed micronuclei (Figures 2H,I), leading to meiotic elimination of chromosomes including, in this case, the *T. ambiguum*-derived chromosome.

### Molecular Cytogenetic Analysis of BC_1_-120 Somatic Cells

**Figure 3:**
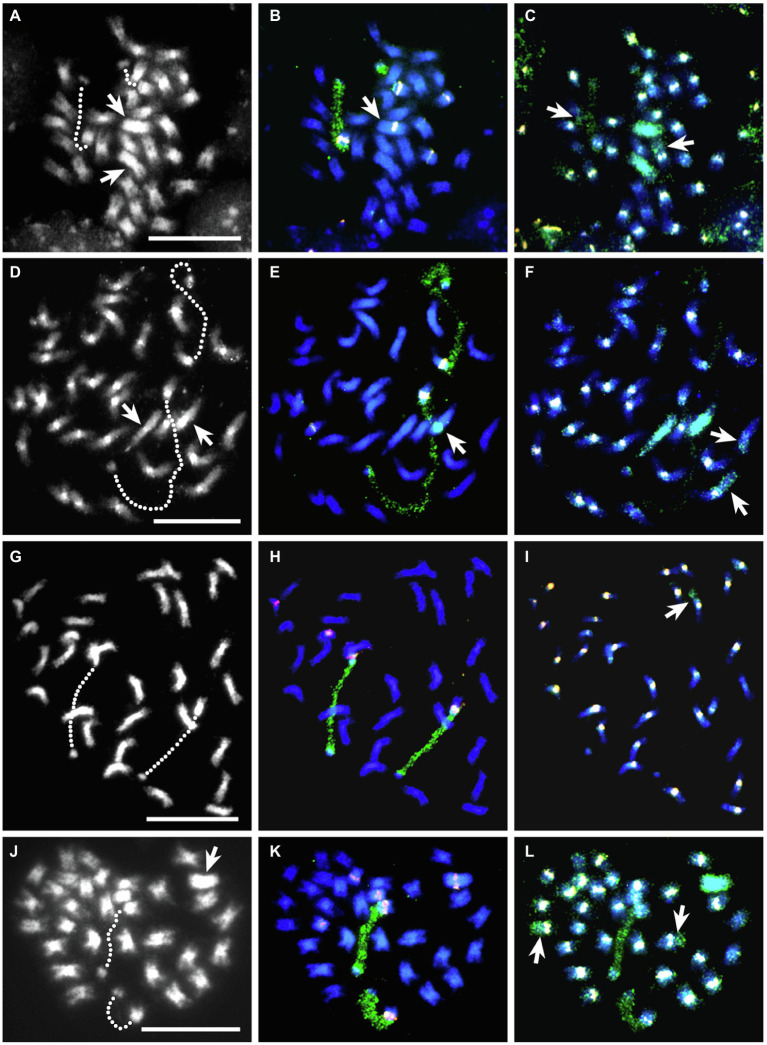
FISH-GISH results for somatic cells of four interspecific hybrids. The left column is DAPI stained (gray scale), the center column the same cell with 5S rDNA (pink) and 18S rDNA (green) FISH probes and the right column the same cell with sequential FISH-GISH using the centromeric probe TrR350 (white) and *T. ambiguum* genomic DNA (green). Hatched lines in the left column show the decondensed NOR-regions of each pair of NOR-chromosomes from *T. repens* or *T. occidentale* that show as green 18S rDNA signals in the middle column. **(A–C)**, BC_1_-120; in **(A)**, the arrows show two large *T. ambiguum* chromosomes that were apparent following GISH in **(C)**. In **(B)**, the arrow indicates that one of the *T. ambiguum* chromosomes carried a 5S rDNA signal. In **(C)**, the arrows mark recombinant chromosomes, one of which lacks a TrR350 centromeric signal. **(D–F)**, BC_1_-132; in **(D)**, the arrows show two large *T. ambiguum* chromosomes that were apparent following GISH in **(F)**. In **(E)**, the arrow shows that one of the *T. ambiguum* chromosomes carried a condensed NOR region (detected by the 18S rDNA probe). In **(F)**, the arrows mark recombinant chromosomes, one of which has a very weak TrR350 centromeric signal. **(G–I)**, Kopu II × BC_1_-132; in **(I)**, the arrow shows a recombinant chromosome. **(J–L)**, BC_2_-133; in **(J)**, the arrow shows a large *T. ambiguum* chromosome which is visible in **(L)** and carries a 5S rDNA signal **(K)**. In **(L)**, the arrows show two recombinant chromosomes, both with strong centromeric TrR350 signals. Bars 10 μm.

Somatic cells of BC_1_-120 showed 33 chromosomes when DAPI-stained, including two very large chromosomes (Figure 3A). These proved to be *T. ambiguum*–derived as shown by GISH using genomic DNA from *T. ambiguum* (Figure 3C). GISH also identified two recombinant chromosomes: one with centromeric TrR350 and with most of one arm consisting of *T. ambiguum* DNA and the other lacking TrR350 and having *T. ambiguum* DNA spanning the centromeric region (Figure 3C). FISH using the TrR350 probe also gave pericentromeric signals on all except three *T. repens* and *T. occidentale* chromosomes and the *T. ambiguum* chromosomes. FISH using the 18S rDNA probe labeled the 18-26S rDNA regions of the NORs which were highly decondensed and spanning chromosome segments that otherwise appear to be unattached (Figures 3A,B). FISH using the 5S rDNA probe produced signals on six chromosomes. One was on the long arm of one of the *T. ambiguum* chromosomes (Figures 3B,C) and two were on two *T. repens* or *T. occidentale* NOR chromosomes with 18-26S rDNA on the opposite arms. The remainder were also on *T. repens*- or *T. occidentale*-derived chromosomes (Figures 3B,C).

### Chromosome Pairing Analysis in BC_1_-120

Conventional cytogenetic analysis of chromosome pairing in BC_1_-120 revealed that an average of just over half the chromosomes paired as bivalents and there were significant numbers of univalents and multivalents including a very low frequency of apparent pentavalents (Vs; Table 4).

### Molecular Cytogenetic Analysis of Somatic Cells of BC_1_-132 and a Derived BC_2_ Plant

Hybrid BC_1_-132, with different parents from BC_1_-120 (Table 2), was also subjected to FISH and sequential FISH-GISH treatments. This hybrid had 33 chromosomes. GISH using *T. ambiguum* genomic DNA revealed the presence of two large *T. ambiguum* chromosomes and two recombinant chromosomes, each apparently with one *T. ambiguum* arm (Figures 3D–F). FISH with TrR350 showed that one of the recombined chromosomes was clearly labeled in the pericentromeric region while the other showed only a very minor signal (Figure 3F). FISH using the 5S and 18S rDNA probes identified two *T. repens* or *T. occidentale* chromosomes with decondensed NORs (Figures 3D,E). One of the *T. ambiguum* chromosomes carried an 18-26S rDNA (NOR) locus that was condensed in all observed cells (Figures 3E,F). There were also two *T. repens* or *T. occidentale* chromosomes with 5S signals (Figures 3E,F).

BC_1_-132 was backcrossed as male to a white clover plant Kopu II-905 and one BC_2_ progeny plant (Kopu II-905 × BC_1_-132)-6 was subjected to FISH and sequential FISH-GISH procedures. This plant was highly fertile (80% pollen staining) and had 32 chromosomes. GISH showed that there were no complete *T. ambiguum* chromosomes, but *T. ambiguum* DNA occurred as one full arm of a single recombinant chromosome. This chromosome had TrR350 in the centromeric region (Figure 3I). FISH with the rDNA probes revealed, as in BC_1_-132, two *T. repens* or *T. occidentale* chromosomes with decondensed NORs and two further *T. repens* or *T. occidentale* chromosomes with 5S signals (Figures 3G–I).

### Molecular Cytogenetic Analysis of BC_2_-133 Somatic Cells

The analysis of a third BC_2_ hybrid plant (BC_2_-133) is presented in Figures 3J–L. Among the 32 chromosomes there was one *T. ambiguum* chromosome with a 5S signal (Figures 3J,K), and two NOR chromosomes and three 5S chromosomes from *T. repens* or *T. occidentale* (Figures 3J,K). Two recombinant chromosomes are shown, both apparently with nearly whole *T. ambiguum* arms attached to TrR350 labeled centromeres (Figure 3L).

### Hybrid Plant Phenotypes

Plant morphology traits were measured in separate outdoor experiments for a small sample of plants in each of the BC_1_ and the BC_2_ and BC_1_F_2_ generations (Supplementary Material). In general, the BC_2_ plants were more white clover-like than the BC_1_ plants, and the hybrid populations were highly variable in phenotype and contained plants with good vigor and fertility while combining traits from the parent species. No rhizomes were observed as these take up to 18 months to develop and the experiments were of short duration.

**Figure 4:**
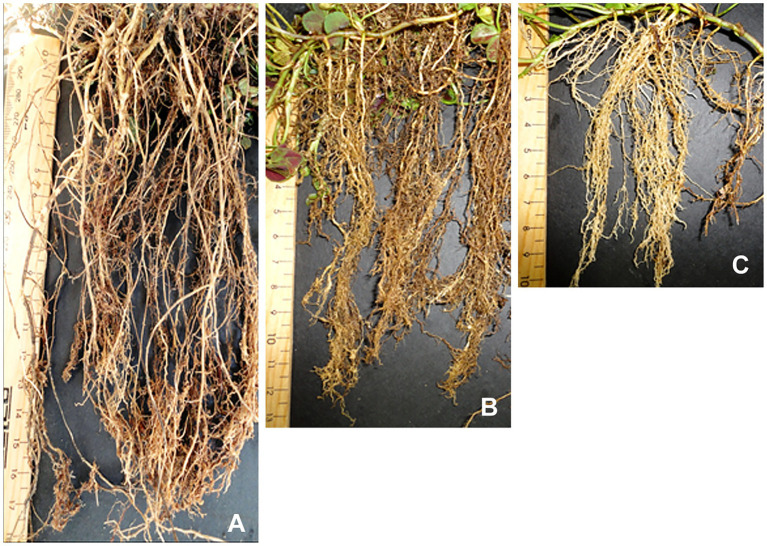
Root morphology of BC_2_-126 **(A)** as compared with control white clover genotypes colored white clover, PxB **(B)** and Kopu II **(C)**. The root system in BC_2_-126 was thicker and deeper compared to the white clover parents.

Figure 4 compares the root systems of hybrid BC_2_-126 with two white clover plants. The hybrid (Figure 4A) showed sparse thick nodal roots while the white clovers both had a concentration of fine nodal roots (Figures 4B,C).

## Discussion

In the present study we tested the hypothesis that recombination between *T. ambiguum* and *T. occidentale* chromosomes can occur and the recombinant chromosomes can be transmitted by hybridization to *T. repens*. White clover-like plants with some *T. ambiguum* traits were produced and, in all five backcross hybrids that were studied in detail at the chromosome level, there was molecular evidence of *T. ambiguum* introgression through chromosomal recombination. This is the first report of chromosomal recombination involving these species, and the results experimentally confirm the hypothesis. This opens the way for the breeding of new forms of white clover incorporating parts of the of *T. ambiguum* genome by using *T. occidentale* as a bridging species.

To-date, only first and second backcross generations have been analyzed and these are still genomically unrefined. All but one of the analyzed plants still carried whole *T. ambiguum* chromosomes and, in addition to recombinant chromosomes, there were unresolved chromosome substitutions, deletions and additions. These can be expected to be resolved by further backcross breeding and selection. Despite the genomic imbalances, the hybrids from F_1_ onward were fertile and able to produce both viable pollen and seeds in sufficient numbers to enable large numbers of progeny to be produced by normal pollination.

The F_1_ parent plants listed in Table 1 (putatively P^r^O^r^AO) were, with one exception, likely to have been true to expectation. However, the AAOO parent of these plants (BL-1) was shown to have 15–17 disjunction at meiosis I in about 10% of PMCs (Williams et al., 2019a), and so some aneuploidy was probable. One F_1_ parent with a flow cytometric estimate of 3.9 was confirmed as 2*n* = 32. The remainder, with estimates of 3.8–3.9 were also probably euploid. However, F_1_-124, had an estimate of 3.6 and may have been aneuploid.

### Molecular Cytogenetic Analysis of Somatic Chromosomes of the Hybrids

In conventional cytological preparations, the somatic chromosomes of *T. ambiguum*, *T. repens*, and *T. occidentale* were small and metacentric to sub-metacentric (Ansari et al., 1999), and so, apart from the NOR-chromosomes, the individual chromosome pairs were indistinguishable from one another within genomes. The chromosomes of *T. ambiguum* were notably larger than those of the other species and could often be recognized by their size in conventional somatic cell preparations of hybrids (e.g., Figures 3A,J).

The use of 5S and 18S rDNA probes together enabled identification of two chromosome pairs in each of *T. repens*, *T. occidentale*, and *T. ambiguum* (Ansari et al., 1999). The NOR-chromosomes of *T. repens* and *T. occidentale* both have a 5S signal on the long arm opposite to the NOR on the short arm (Figures 3B,E,H,K) and could not be distinguished from each other. However, the *T. ambiguum* NOR-chromosome could be identified as it lacked a 5S signal (Figures 3E,F). The 5S rDNA probe could detect one further chromosome pair from *T. repens* (a single large signal on the long arm) and one from *T. occidentale* (a minor signal on the short arm) while the remaining six chromosome pairs of both species remain unmarked and indistinguishable (Ansari et al., 1999). In the present study, resolution of these markers was variable, and no attempt was made to distinguish between the 5S signals from *T. repens* and *T. occidentale* in the hybrid genomes. *T. ambiguum* also had a distinctive chromosome pair with a 5S locus (Figures 3B,C,K,L). Use of the FISH probe TrR350 (Ansari et al., 2004) could usefully distinguish most *T. ambiguum* chromosomes (one pair with TrR350) from *T. repens* (all chromosomes) and *T. occidentale* (all or most chromosomes).

Application of the GISH technique to the hybrid genomes using a *T. ambiguum* genomic DNA probe was potentially able to detect the presence of any part of the *T. ambiguum* (A) genome, including intact A chromosomes and chromosomes involving A recombinations.

### Aneuploidy and Chromosome Imbalances

All the analyzed BC_1_ and F_2_ progeny derived from the F_1_ parents were aneuploid (Table 2). Three BC_1_ and one F_2_ plant derived from a single F_1_ (F_1_-119) varied both up and down from the expected 2*n* = 32 (Table 2). Thus, whatever the parental chromosome number, all the gametes involved in producing the analyzed BC_1_s from this plant varied from the expected *n* = 16 (The other parent, *T. repens*, reliably produces euploid *n* = 16 gametes). Meiotic chromosome pairing was not studied in the P^r^O^r^AO hybrids, but genomically balanced gametes could not occur, and it is highly likely that several chromosomes (especially A chromosomes) would have been unpaired at meiosis, leading to aneuploid gametes. Consequently, progeny plants would have had unpredictable numbers of chromosomes from each of the four contributing sub-genomes. Thus, while the average expected gametic constitution would have been P^r^_4_O^r^_4_A_4_O_4_, it was apparent that none of the tested BC_1_ plants were derived from such gametes, and that chromosome additions, deletions and substitutions were inevitable in the following generations. This was supported by meiotic pairing analysis of one of these plants (BC_1_-120, Table 4), where univalent and multivalent meiotic chromosome configurations were frequent. In the next generation, the moderately fertile BC_2_ plant with two introgressed segments from *T. ambiguum* (BC_2_-126, 2*n* = 33) had one full *T. ambiguum* chromosome and 32 combined *T. occidentale* and *T. repens* chromosomes. However, these 32 were not a balanced set as evidenced by the presence at meiosis in this plant of more than one univalent, on average, as well as trivalents (Table 4). All three BC_2_ plants had 32 or 33 chromosomes and moderate to high fertilities (Table 3), suggesting that any sub-genomic imbalances did not drastically reduce fertility. A high frequency of asynapsis (i.e., univalents) at meiosis can be associated with the generation of 2*n* gametes in interspecific hybrids (Ansari et al., 2022). However, in the materials analyzed, no products of 2*n* gametes were observed.

FISH-GISH analyses of BC_1_-120 and BC_1_-132 showed that, in both cases, there were 33 chromosomes, but with only two rather than the expected four A chromosomes (Table 5). The A chromosomes had apparently been lost at a greater rate than expected, probably due to lack of pairing partners at meiosis I. The presence of 33 chromosomes indicated that chromosomes derived from *T. repens* and *T. occidentale* had more than substituted for the lost *T. ambiguum* chromosomes.

**Table 5:** Summary of the results of FISH-GISH analyses of five interspecific hybrid plants using 18S rDNA (NOR), 5S rDNA (5S), *T. ambiguum* genomic DNA (A), and TrR350 probes.

	Number of chromosomes					
	Total 2*n*	R or O NOR	R or O 5S non-NOR	A	Recombinant chromosomes	
Hybrid plant					Total	Without TrR350
BC_1_-120	33	2	2	2	2	1
BC_1_-132	33	2	2	2	2	0
BC_2_-126	33	2	3	1	2	NA
Kopu II × BC_1_-132	32	2	2	0	1	0
BC_1_-133	32	2	3	1	2	0

*NA, not analyzed*.

Consistent with the apparent rapid loss of A chromosomes, the analyzed BC_2_ plants had one or no A chromosomes instead of the expected two (Table 5). Meiotic analysis of BC_2_-126 showed that, nearly 50% of the time, the A chromosome was unpaired at meiosis and so was likely to be lost by unequal disjunction from half of the gametes. In addition, it appeared that A univalents sometimes underwent premature disjunction (separation of sister chromatids) at anaphase I, followed by incomplete movement to the poles (lagging; Figures 2F–I). It is probable that this further exacerbated the loss of the A chromosome.

Non-NOR chromosomes with 5S loci also revealed chromosome imbalances in both BC_1_ and BC_2._ On average, one such pair was expected, as in *T. repens* and *T. occidentale*, but with potential variation from 1 to 3, assuming random assortment in the previous generation. The BC_1_ and BC_2_ plants had two or three such chromosomes (Table 5).

### Meiotic Analyses and Recombinant Chromosomes

In this study, GISH signals that were consistently visible in multiple cells were regarded as definite signals. There were several cases in which small recombinant segments were apparent in a few cells but were not definite enough to confirm. Consequently, the most consistently visible sectors have been recorded, but this a minimum estimate and there may have been more recombination than identified here. Nevertheless, it was significant that recombinant chromosomes were found in all five analyzed BC plants and that four plants had at least two recombinant chromosomes. Therefore, the inter-genomic recombination frequency was significant and sufficient to encourage further development of an introgression breeding program.

Despite aneuploidy, the BC_1_ plants had pollen staining of 30–67% indicating reasonable fertility and production of functional gametes. Leading up to the generation of the BC_1_ hybrids, there had been two opportunities for pairing and recombination between A and O sub-genomes (Figure 1). First, in the AAOO hybrid used to generate the P^r^O^r^AO plant there could have been A-O recombination leading to gametes with *T. occidentale* chromosomes carrying *T. ambiguum* segments and vice versa (Williams et al., 2019a). Second, in the P^r^O^r^AO hybrid there was further opportunity for interchanges among A and O and perhaps A and O^r^ chromosomes and, also, P^r^ and O^r^ and O chromosomes, with potentially some of those chromosomes carrying A segments.

In the BC_1_ plants there would also have been further opportunities for inter-species pairing and recombination. Analysis of BC_2_-126 showed that after two backcrosses the genomes had not regained balance and departed significantly from a stable pattern of 16 bivalents and one univalent produced by the lone A chromosome. Instead, univalent frequency was 3.5 (range 0–9), indicating that R and O chromosomes were also unbalanced, probably lacking homologs or competing for homologs, and not regularly pairing. The presence of multivalent configurations was indicative of homoeologous pairing. The absence of univalents in some cells (Table 4) was indicative that the *T. ambiguum* chromosome sometimes paired with *T. repens* or *T. occidentale* chromosomes, and this was confirmed by FISH-GISH of meiosis which showed it to be paired in over 50% of the cells studied. The recombinant chromosomes were also shown to pair with *T. repens* or *T. occidentale* chromosomes (Figure 2E). Thus, further backcross progeny from this and the other BC_2_ plants could potentially have led to further recombination and integration of *T. ambiguum* into a predominantly white clover genetic background. This is supported by the finding that, despite the presence of recombinant chromosomes and the potential genomic imbalances, BC_2_-126 was moderately fertile and able to produce large numbers of progeny for further breeding (Supplementary Table 2B).

The R sub-genomes (P^r^ and O^r^) never pair at meiosis in white clover, probably because of a genetic system preventing homoeologous pairing (Williams, 2014). However, they do pair in interspecific hybrids, as shown in 4*x T. repens* × *T. uniflorum* and 4*x T. repens* × *T. occidentale* hybrids (Hussain et al., 2016, 2017; Hussain and Williams, 2016). Probably they could also pair with each other in the P^r^O^r^AO hybrids, leading to novel P^r^-O^r^ recombination. A and O chromosomes also show pairing affinities (Williams et al., 2011, 2019a), enabling A-O recombination. P^r^-O and O^r^-O pairing is also likely (Hussain and Williams, 2016), enabling introgression of *T. occidentale* into a white clover background. On the other hand, as already noted, in *T. ambiguum* × *T. repens* hybrids the A and R sub-genomes have shown very poor pairing affinity, suggesting that P^r^-A and O^r^-A pairings occur infrequently in those hybrids. The reason why O^r^-A pairing is apparently much less frequent than O-A pairing has not been investigated but could be related to the restriction of homoeologous pairing in *T. repens* (Williams, 2014).

Of the recombinant chromosomes identified in BC_1_ and BC_2_ plants, all except one involved a *T. ambiguum* segment attached to a *T. occidentale* chromosome, as identified by the centromeric probe, TrR350 (Table 5). The one exception occurred in hybrid BC_1_-120, where one of the recombinant chromosomes had a *T. occidentale* segment attached to a *T. ambiguum* centromere. Here, the centromere lacked TrR350 and the *T. ambiguum* DNA signal spanned the centromeric region (Figure 3C). This difference in the frequencies of the recombinant chromosome classes might reflect the numerical presence of two *T. occidentale* (O and O^r^) genomes and one only *T. ambiguum* genome in the F_1_ hybrids. Alternatively, or in addition, there was lower than expected transmission of *T. ambiguum* chromosomes, as already noted.

The BC_2_ plant, Kopu II × BC_1_-132, although probably still unbalanced, came closest among the analyzed plants to achieving the aims of the backcross program. This plant had 32 chromosomes, including one with a large translocated segment from *T. ambiguum*. Otherwise, it had no additional A chromosomes and showed the desired numbers of NOR chromosomes and 5S carrying chromosomes from *T. repens* or *T. occidentale*. Further backcrossing would be expected to produce families with large numbers of plants of this type, with near-balanced karyotypes and potential for achieving the introgression-breeding aims.

### Epigenetic Interactions

BC_1_ plants consistently had a pair of NOR chromosomes each with a 5S locus, as in *T. repens* and *T. occidentale*. In all cases these NORs were active (decondensed). Hybrid BC_1_-132 had an extra A chromosome carrying an NOR region (Figures 3E,F) which, by contrast, was inactive (condensed) in all observed cells. This nucleolar dominance (Chen and Pikaard, 1997) reflects epigenetic suppression of the *T. ambiguum* derived NOR locus. Similar suppression of the *T. ambiguum* NOR was observed in diploid *T. ambiguum* × *T. occidentale* hybrids (Williams et al., 2011) and some other *Trifolium* interspecific hybrids (Williams et al., 2019b). However, other hybrids, e.g., BL-1, the 4*x* hybrid parent of the present study, showed activity (decondensation) of both *T. ambiguum* and both *T. occidentale* NORs (Williams et al., 2019a). A concern for breeding by incorporation of genes from one species into another is whether the expression of donated genes will be epigenetically suppressed by the host genome (Adams et al., 2003). In the present case, the expression of *T. ambiguum* traits in the backcross hybrids suggested that this might not be a problem with these hybrids. However, in all but one BC hybrid there were remnant A chromosomes and it remains to be tested whether introgressed *T. ambiguum* traits will be fully expressed in advanced generations once whole A chromosomes have been eliminated.

### The Consequences of Substitution of O^r^ by O-A Recombinant Chromosomes

The original proposition for this work invoked a two-stage transfer of *T. ambiguum* DNA to *T. repens* chromosomes *via* a *T. occidentale* chromosome bridge. However, recent DNA sequence analyses of *T. repens* and its two progenitors, *T. occidentale* and *T. pallescens* have revealed that the ancestral *T. occidentale* (O^r^) sub-genomes currently in *T. repens* are largely unchanged from the genomes of extant *T. occidentale* (Griffiths et al., 2019). This suggests that substitution of O^r^ sub-genome chromosomes by O chromosomes from *T. occidentale* might have very little disruptive effect on genome functions. If so, then attachment of *T. ambiguum* segments to a predominantly *T. occidentale* chromosome and the substitution of this hybrid chromosome for its homolog in white clover by backcrossing could achieve introgression without the need for the second crossover into the white clover homolog. This requires only A-O crossover events followed by chromosome substitution during backcrossing and removes the need for a final R-A/O recombination event.

### The Significance for Clover Breeding

The hybrids represent a potentially powerful breeding resource for extending the white clover gene-pool. First, the chromosome pairing patterns in these hybrids are of special significance because of their potential for novel genomic recombinations leading to introgression. Second, as already noted, they provide potential for chromosome substitutions, additions and subtractions. A breeding program involving large populations of interspecific hybrids has been established for the purpose of incorporating *T. ambiguum* traits into white clover.

The selection strategy used in the present study was based on above-ground traits of BC hybrid plants for expression of *T. ambiguum* traits along with those of the recurrent parent, *T. repens*. Even though this would have favored selection of plants with remnant A chromosomes, all the tested plants carried recombinant chromosomes, and one carried no A chromosomes. Although only a small sample, this would tend to suggest that recombination frequencies were high, and enough to provide confidence that this introgression strategy is workable. To-date, there has been no testing for resilience traits such as drought tolerance (which requires complex time-consuming experiments), or rhizome development (which take up to 18 months to develop). These tests will be needed in later generations now that the introgression strategy is viable.

As noted earlier, *T. ambiguum* exists in 2*x*, 4*x* and 6*x* forms, each with distinctive but overlapping ecological adaptations. The breeding scheme described (Figure 1), and tested here, used a 4*x* form of *T. ambiguum*. However, we have previously shown that a colchicine-doubled form derived from 2*x T. ambiguum* showed similar behavior in hybrids with *T. occidentale* (Williams et al., 2019a). It is probable that the scheme can be used to incorporate genetics from both 2*x* and 4*x T. ambiguum*, thus widening the pool of adaptations available for incorporation into *T. repens*. The 6*x* form has also been incorporated into three-way hybrids with *T. occidentale* and *T. repens* (Williams et al., 2019b) but to-date, these have not been developed. The early generation hybrids were near-6*x* due to reliance on unreduced gametes (2*n* = 4*x*) and several generations of further breeding may be necessary to produce white clover plants carrying 6*x T. ambiguum* genes (Williams et al., 2019b).

The progressive and relatively rapid loss of A chromosomes was desirable for an introgression breeding strategy. While some A chromosomes were present for a few generations, facilitating recombination, their relatively rapid disappearance should contribute to a timely return to a *T. repens* genomic background and the production of plants that are essentially *T. repens* integrated with short genomic segments from *T. ambiguum*. It was also notable that different A chromosomes were retained in some of the analyzed BC hybrids, indicating no evidence to-date of any undesirable retention of particular A chromosomes.

Three of the plants reported in Table 3 were the progeny from self-pollination of BC_1_ hybrids. These were expected to retain some *T. ambiguum* chromosomes and *T. occidentale* chromosomes in a near-4*x T. repens* genetic background. The rationale for producing these selfs was to move toward the production of *T. ambiguum* substitution and/or addition lines in which pairs of *T. ambiguum* chromosomes stably replace or supplement *T. repens* chromosomes. Such stable lines could be expected to further enhance clover breeding by providing an alternative strategy to transfer the desirable resilience traits from *T. ambiguum* into *T. repens*. These hybrids produced progeny for further study (Supplementary Table 2B).

These new results contrast with earlier attempts to combine the favorable traits of 4*x T. ambiguum* into white clover which have not revealed introgression by recombination. A strategy based on backcrossing 4*x T. ambiguum* × *T. repens* F_1_ hybrids to *T. repens* was developed by Meredith et al. (1995). As outlined earlier, this strategy required transitions from 4*x* (F_1_) to 6*x* (BC_1_) and then back to 5*x* (BC_2_) and on through presumed aneuploid generations (BC_3_ onwards) and, finally, 4*x* in later generations. Expression of rhizome formation (Abberton et al., 1998, 2003) and drought tolerance (Marshall et al., 2001, 2015) were reported up to the BC_3_ generation when aneuploidy, potentially involving the addition or substitution of several whole *T. ambiguum* chromosomes into the *T. repens* genome, was expected (Abberton et al., 2003). Reports of the phenotypes of selected populations beyond the BC_2_ generation have not included any chromosome numbers or evidence of introgression *via* chromosome recombination. There is very limited pairing between *T. ambiguum* and *T. repens* chromosomes (Williams et al., 1982; Anderson et al., 1991; Meredith et al., 1995) and to-date there is no evidence of meiotic recombination leading directly to introgression of *T. ambiguum* segments into *T. repens* chromosomes. Nevertheless, as noted, chromosome additions or substitutions could occur instead. Although the details are unclear, the strategy produced the commercially sold interspecific hybrid (cv. ‘Aberlasting’) with some *T. ambiguum* traits. By contrast, the breeding strategy developed here achieved introgression by recombination and avoided higher ploidy (6*x* and 5*x*) generations.

To achieve the breeding objective of attaining white clover populations incorporating genes for greater resilience from *T. ambiguum*, further steps are needed. First, because the BC_2_ plants had slightly unbalanced genomes, further backcrosses (with selection) are needed to obtain stable families with the desired phenotypes. This needs to be applied to large numbers of BC plants from diverse families to build a gene-pool of introgressed white clover families each carrying different arrays of introgressed material. Then new cultivars will be developed by selection for the desired resilient phenotypes using the full arsenal of available breeding methods. The molecular cytogenetic methods used here will probably play a useful but minor role in large scale breeding. Other genomic methods will have a major role for all traits, and especially for those that are difficult to measure, e.g., rhizomes, deep roots, drought tolerance; as applied for example by Abberton et al. (2003) to selection for rhizomes. Knowledge of the genomes of *T. repens*, *T. pallescens* and *T. occidentale* (Griffiths et al., 2019) will facilitate the unlocking of the *T. ambiguum* genome and enable the use of genomic methods to further track *T. ambiguum* introgressions against the *T. repens* background. Determinations of the breeding values of each introgression through genotyping and phenotypic characterization should provide rapid genetic gains in future clover breeding.

## Data Availability Statement

The original contributions presented in the study are included in the article/Supplementary Material, further inquiries can be directed to the corresponding author.

## Author Contributions

IU carried out the experiments, analyzed the results and wrote part of the manuscript as part of a PhD degree program. HA performed and interpreted the molecular cytogenetic work. IV conceived the breeding strategy, carried out the interspecific hybridization and provided advice throughout the project. SH provided guidance for the conventional cytogenetics. NE carried out the molecular biology work and supplied the probes used for molecular cytogenetics. MM co-supervised the PhD component. WW led the project and co-wrote the manuscript. All authors contributed to the article and approved the submitted version.

## Funding

The research was funded primarily by the NZ Ministry of Business, Innovation and Employment Grant C10X0711 with support by PGG-Wrightson Seeds. IU gratefully acknowledges financial assistance from the Higher Education Commission of Pakistan and overseas study leave from the Pakistan Agricultural Research Council. AgResearch and Massey University funded the publication costs.

## Conflict of Interest

The authors declare that the research was conducted in the absence of any commercial or financial relationships that could be construed as a potential conflict of interest.

## Publisher’s Note

All claims expressed in this article are solely those of the authors and do not necessarily represent those of their affiliated organizations, or those of the publisher, the editors and the reviewers. Any product that may be evaluated in this article, or claim that may be made by its manufacturer, is not guaranteed or endorsed by the publisher.
