# Supplementary material for: Introgression of Trifolium ambiguum Into Allotetraploid White Clover (Trifolium repens) Using the Ancestral Parent Trifolium occidentale as a Bridging Species
Source: Front Plant Sci. 2022 Mar 18;13:858714. doi: 10.3389/fpls.2022.858714 (PMC8971954; doi:10.3389/fpls.2022.858714)
Supplement: Supplementary file 1 [file Data_Sheet_1.docx]

**Supplementary materials**

**Morphological descriptions of the hybrids**

***Methods***

Selected first and second backcross hybrids and controls (listed in Tables S1 and S2) were planted in two separate outdoor trials. The first was planted on 5-12 May 2010 and the second on 6-8 June 2011 at the Grasslands Research Centre, Palmerston North, New Zealand. This is a temperate site (mean annual rainfall 917.3 mm, annual sunshine hours 1743.5, mean temperature 13.1°C., mean daily maximum 17.8°C., mean daily minimum 8.5°C). For these experiments, clonal cuttings were obtained from the plants selected to be included in the experiment. Care was taken that the cuttings were of the same size and physical condition. Each cutting consisted of 3-4 cm of stem with one active growing point and one main root with three fully opened trifoliolate leaves. Initially these were planted in plastic pots of 8 cm diameter filled with 1:1 sand and peat potting mix in a glasshouse with natural day length and a temperature range of 15-25°C. The pots were watered as needed and a complete soluble fertilizer (Yates Thrive®) was applied fortnightly to maintain soil fertility. After one month the plants were replanted outside in a sandpit with a sand depth of 45 cm where both experiments were laid out in randomized complete block designs with four replicates. Each plant was allocated an area of 0.36 m^2^. They were watered daily, usually in the evening, and more intensively in the summer months to maintain sub-surface moisture, and fed with a complete soluble commercially available plant nutrient supplement (Yates Thrive®) on a weekly basis at the rate of 250 mL per plant. Morphological observations on the first experiments containing BC_1_ hybrids were recorded on the above-ground qualitative and quantitative traits in January 2011. During May 2011, this experiment was harvested destructively by digging up the plants and data were recorded on above- and under-ground components of the plants. Similarly, data on the second trial were recorded during January and February 2012. Following destructive harvests, plant components were oven-dried at 80°C for 48 hours and then the dry weights of above-ground parts, root system and total biomass were recorded using an electronic scale. The traits on which data were collected are given in Tables S1 and S2. Leaf measurements were taken on a typical first fully opened leaf from the stolon tip. Petiole lengths were an average of five random petioles per plant. Stem thickness was measured in the centre of a typical internode using Vernier electronic calipers. Stem anchoring was recorded on a 0-10 scale (0, no nodal roots, 10 all nodes rooted as in white clover). Root thickness was measured with Vernier calipers 1 cm from the attachment to the stem. Flower and seed data were collected from a typical inflorescence (head) per plant. Where tests for normality or homogeneity of variances indicated the need, data were transformed as indicated in Tables S2a and S2b. Analysis of variance (ANOVA) was carried out by using Genstat 12th edition (Payne *et al*., 2010).

**Results**

*BC_1_ hybrids*

Four BC_1_ hybrids and one F_2_ plant were evaluated in the first field experiment, along with the parent F_1_ (Red-1 x BL-1)-19 and the red-leaved PB17 white clover control (Tables S1a, b). Significant differences among the plants were revealed for all traits, reflecting the marked phenotypic variation generated. The F_2_ plant was semi-stoloniferous with nodal roots only at the basal two to three nodes after which the stems were semi-erect to erect without nodal roots. The growth habit was determinate *(T. ambiguum*-like) in the F_1_ parent and the F_2_. The F_1_ and F_2_ plants were smaller in most above-ground traits and had fewer seeds per head than the white clover control. However, both had higher ratios of root to total dry weight, reflecting *T. ambiguum* influence. All the BC_1_ hybrids developed horizontal, nodally-rooted stolons, but they were less well anchored than white clover. The growth habit showed determinate growth (failed or very slow growth of the terminal meristem) associated with axillary flowering. All the BC_1_ hybrids were female-fertile but the F_2_ did not set seed after open pollination. Two of the four BC_1_ hybrids were larger in terminal dry weights than the white clover control and root weight % of the total biomass was significantly higher than the white clover control in one BC_1_.

*BC_2_ and BC_1_F_2_ hybrids*

BC_2_ and BC_1_F_2_ hybrids were evaluated in the second field trial along with a single F_2_-derived BC_1_ plant and two white clover control plants. The BC_2_ hybrids, with a few exceptions, were more similar to white clover than the BC_1_ hybrids (Tables S2a, b). The BC_2_ hybrids formed stolons with indeterminate apical growth and axillary flowering as in white clover, but the level of nodal rooting (anchorage) was significantly lower than white clover in all but one hybrid. The F_2_-derived BC_1_ plant (BC_F2:1_-136) was non-stoloniferous and had determinate growth in combination with axillary flowering. The BC_2_ and BC_F2:1_ plants were compared with the commercial white clover Kopu II control plant as this was most similar to the second backcross parents. They were smaller for almost all traits but all the hybrids except one had significantly higher root to total yield ratio (17-49%) than Kopu II (11%).

Two BC_1_F_2_ plants were compared with the red-leaved white clover control plant (PB1 x PB2)-2, which was closely related to the predominant first backcross parents. Nodal rooting (anchorage) was poorer than white clover while one of the BC_1_F_2_ plants was larger than the control for several above-ground traits. Flowering, where it occurred, was axillary and fertility traits (florets/head and seeds/head) were similar to white clover. For unknown reasons, three of the four ramets of one clone failed to flower while the fourth showed flowering similar to white clover.

**Conclusion**

Overall, the results of this analysis of a small sample of plants in each hybrid generation have demonstrated that the introgression breeding program generated hybrid populations that were highly variable in phenotype and contained plants with good vigour and fertility while combining traits from the parent species. The results revealed a need to continue backcrossing beyond BC_2_ to restore full nodal rooting as in white clover. A key trait, rhizome development, was not assessed because this requires longer duration trials of at least 18 months.

**Table S1a.** Mean morphological data of the above-ground traits of four BC_1_ and one F_2_ plant with the F_1_ and white clover parents as controls.

| **Plant** | **Stolon length (cm)** | **Stolon no.** | **Stem anchorage (0-10)** | **Head no.** | **Peduncle length (cm)** | **Florets/head** | **Leaflet length/width ratio** | **Petiole length (cm)** | **Flowering**  **terminal,**  **axillary or combination** |
| --- | --- | --- | --- | --- | --- | --- | --- | --- | --- |
| **BC_1_-120** | 24.4 | 27.7 | 8.5 | 148 | 17.4 | 67.4 | 1.32 | 13.5 | combination |
| **BC_1_-128** | 67.3 | 71.3 | 9.3 | 605 | 19.9 | 49.9 | 1.45 | 11.6 | combination |
| **BC_1_-130** | 58.7 | 41.5 | 7.5 | 176 | 16.1 | 45.3 | 1.57 | 9.2 | axillary |
| **BC_1_-132** | 53.4 | 68.8 | 8.0 | 425 | 19.3 | 59.4 | 1.25 | 11.2 | combination |
| **F_2_-133** | 6.0 | 13.2 | 0.8 | 47 | 10.0 | 34.5 | 1.88 | 4.4 | combination |
| **F_1_ (Red-1 x BL-1)-19** | 15.9 | 20.0 | 7.3 | 78 | 12.0 | 77.7 | 1.53 | 4.4 | combination |
| ***T. repens* PB17** | 29.8 | 35.0 | 10.0 | 83 | 13.6 | 64.6 | 1.44 | 9.7 | axillary |
| **SEM** | 2.04 | 3.15 | 0.48 | 26.2 | 1.14 | 2.29 | 0.045 | 1.43 |  |
| **LSD** | 6.07 | 9.37 | 1.43 | 78.0 | 3.39 | 6.80 | 0.14 | 4.25 |  |
| **Prob. (P<0.001)** | ******* | ******* | ******* | ******* | ******* | ******* | ******* | ******* |  |

**Table S1b.** Mean morphological data of the above-ground traits and terminal dry weights of shoots and roots for four BC_1_ and one F_2_ plant with the F_1_ and white clover parents as controls.

| **Hybrids** | **Stem thickness (mm)** | **Seeds/head** | **Shoot dry weight (g)** | **Root dry weight (g)** | **Total**  **biomass (g)** | **Root weight % total biomass** |
| --- | --- | --- | --- | --- | --- | --- |
| **BC_1_-120** | 2.43 | 32.2 | 8.6 | 5.3 | 13.8 | 38.1 |
| **BC_1_-128** | 3.12 | 30.7 | 121.0 | 44.0 | 165.0 | 26.6 |
| **BC_1_-130** | 2.83 | 12.4 | 59.2 | 18.9 | 78.1 | 23.2 |
| **BC_1_-132** | 3.32 | 10.8 | 71.1 | 22.5 | 93.6 | 24.8 |
| **F_2_-133** | 1.99 | 0.0 | 2.0 | 2.5 | 4.5 | 54.5 |
| **F_1_ (Red-1 x BL-1)-19** | 2.51 | 3.7 | 3.9 | 5.7 | 9.6 | 49.2 |
| ***T. repens* PB17** | 2.27 | 37.2 | 26.2 | 7.3 | 33.5 | 23.3 |
| **SEM** | 0.15 | 4.2 | 11.8 | 4.1 | 15.4 | 4.8 |
| **LSD** | 0.46 | 12.6 | 35.0 | 12.2 | 45.7 | 14.1 |
| **Prob. (P<0.001)** | *** | *** | *** | *** | *** | *** |

**Table S2a.** Mean morphological data of the above-ground traits of three BC_2_, two BC_1_F_2_ and one F_2_-derived BC_1_ hybrid plants, with two *T. repens* plants as controls.

| **Hybrids** |  | **Stolon**  **No.** | **Stem**  **Anchorage (0-10)** | **Head**  **No. ‡** | **Florets/head** | **Seeds/head ‡** | **Flowering-terminal, axillary or combination** |
| --- | --- | --- | --- | --- | --- | --- | --- |
| **BC_2_-126** |  | 18.0 | 4.0 | 3.9 (50.5) | 43 | 2.05 (6.8) | axillary |
| **BC_2_-129** |  | 24.3 | 8.0 | 4.4 (81.0) | 51 | 4.32 (74.3) | axillary |
| **BC_2_-131** |  | 10.0 | 5.3 | 3.0 (18.1) | 55 | 3.17 (22.9) | axillary |
| **BC_F2:1_-136** |  | - | 0.0 | 1.5 (3.5) | 25 | 0.41 (0.5) | combination |
| **BC_1_F_2_-137** |  | 22.0 | 6.5 | 3.5 (30.7) | 48 | 3.40 (29.0) | axillary |
| **BC_1_F_2_-140** |  | 7.7 | 6.7 | † | 48† | † | axillary |
| ***T. repens* Kopu II** |  | 48.3 | 10.0 | 6.1 (428.2) | 52 | 3.91 (48.8) | axillary |
| ***T. repens* (PB1 x PB2)-2** |  | 16.0 | 10.0 | 2.7 (14.3) | 48 | 3.23 (24.3) | axillary |
| **SEM** |  | 4.92 | 0.77 | 0.37 | 5.3 | 0.33 |  |
| **LSD** |  | 15.15 | 2.26 | 1.13 | 16.3 | 0.97 |  |
| **Prob. *, ***(P<0.05, 0.001)** |  | ******* | ******* | ******* | ***** | *** |  |

**‡** Data were (ln+1) transformed. Values in parenthesis show back-transformed data.

† Excluded from analysis (only one plant flowered)

- No horizontal stolons formed

**Table S2b.** Mean morphological data of the above-ground traits and terminal dry weights of shoots and roots for three BC_2_, two BC_1_F_2_ and one F_2_-derived BC_1_ hybrid plants, with two *T. repens* plants as controls.

| **Hybrids** | **Stem thickness (cm)** | **Main root thickness (mm)** | **Dry weight**  **top**  **(gms) ‡** | **Dry weight root**  **(gms)‡** | **Total  biomass  (gms) ‡** | **Root weight % total biomass** |
| --- | --- | --- | --- | --- | --- | --- |
| **BC_2_-126** | 3.2 | 2.4 | 2.06 (7.88) | 1.30 (3.67) | 2.45 (11.62) | 32.0 |
| **BC_2_-129** | 2.3 | 2.4 | 3.21 (24.76) | 2.21 (9.16) | 3.53 (33.97) | 27.1 |
| **BC_2_-131** | 2.1 | 2.1 | 1.78 (5.90) | 0.97 (2.63) | 2.16 (8.67) | 31.3 |
| **BC_F2:1_-136** | 2.3 | 2.2 | 0.22 (1.24) | -0.11 (0.89) | 0.77 (2.15) | 41.9 |
| **BC_1_F_2_-137** | 2.3 | 2.2 | 2.38 (10.82) | 0.79 (2.20) | 2.57 (13.03) | 17.0 |
| **BC_1_F_2-_140** | 2.1 | 1.3 | -1.06 (0.35) | -1.10 (0.33) | -0.39 (0.68) | 48.9 |
| ***T. repens* Kopu II** | 3.4 | 2.9 | 5.32 (204.88) | 3.24 (25.46) | 5.44 (231.02) | 11.3 |
| ***T. repens* (PB1 x PB2)-2** | 1.8 | 2.2 | 1.30 (3.65) | 0.47 (1.60) | 1.66 (5.28) | 30.7 |
| **SEM** | 0.14 | 0.26 | 0.34 | 0.35 | 0.34 | 2.73 |
| **LSD** | 0.43 | 0.78 | 1.01 | 1.02 | 0.99 | 8.04 |
| **Prob. *, *** (P<0.05, 0.001)** | *** | * | *** | *** | *** | *** |

**‡** Data were log transformed. Values in parenthesis show back-transformed data.
